# Association between workplace gaslighting, mental health and work life in nurses: a cross-sectional study in Greece

**DOI:** 10.3934/publichealth.2026030

**Published:** 2026-05-11

**Authors:** Aglaia Katsiroumpa, Ioannis Moisoglou, Olympia Konstantakopoulou, Parisis Gallos, Maria Rekleiti, Petros Galanis

**Affiliations:** 1 Clinical Epidemiology Laboratory, Faculty of Nursing, National and Kapodistrian University of Athens, Athens, Greece; 2 Department of Nursing, University of Thessaly, Larissa, Greece; 3 Faculty of Nursing, University of West Attica, Athens, Greece; 4 Andreas Syggros Hospital of Cutaneous and Venereal Diseases, Athens, Greece

**Keywords:** workplace gaslighting, nurses, Gaslighting at Work Scale, anxiety, depression, quiet quitting, work engagement

## Abstract

**Background:**

Workplace gaslighting is an alarming issue. However, the negative consequences of workplace gaslighting in nurses are unknown. In this context, our aim was to examine the association between workplace gaslighting and nurses' mental health and work life.

**Methods:**

We conducted an online cross-sectional study in Greece during December 2024. We employed a convenience sample of nurses. We used the Gaslighting at Work Scale (GWS) to measure levels of workplace gaslighting among our nurses. We used the Patient Health Questionnaire-4, the Quiet Quitting Scale, and the Utrecht Work Engagement Scale-3 to measure anxiety-like symptoms, depressive-like symptoms, quiet quitting, and work engagement, respectively.

**Results:**

The study population included 369 nurses with a mean age of 37.86 years. We found a positive association between workplace gaslighting, anxiety-like symptoms, and depressive-like symptoms in our nurses. After adjustment for confounders, we found a positive association between GWS scores and anxiety-like symptoms (adjusted b = 0.758, 95% CI = 0.606 to 0.909, p < 0.001), and depressive-like symptoms (adjusted b = 0.720, 95% CI = 0.555 to 0.885, p < 0.001). Moreover, our multivariable models showed a positive association between GWS scores and quiet quitting (adjusted b = 0.258, 95% CI = 0.186 to 0.330, p < 0.001). Also, we found a negative association between GWS scores and work engagement (adjusted b = −0.353, 95% CI = −0.512 to −0.195, p < 0.001).

**Conclusions:**

Our findings suggest that nurses who experience higher levels of gaslighting from their supervisors have more anxiety-like symptoms and depressive-like symptoms. Moreover, workplace gaslighting is associated with quiet quitting and work engagement. However, considering the cross-sectional nature of our study and the study limitations, further research should be conducted to extract more valid results.

## 1. Introduction

Gaslighting, often referred to as the gaslight effect, constitutes a form of psychological manipulation typically occurring within dyadic relationships. In such interactions, the individual acting as the gaslighter systematically attempts to impose a particular narrative or interpretation upon the other party, known as the gaslightee [1]. Through repeated strategies of denial, contradiction, and distortion, the gaslighter progressively leads the gaslightee to question his/her own thoughts, actions, and perception of reality. As this process unfolds, the gaslightee's sense of autonomy is weakened, resulting in increasing psychological dependence on the perpetrator [2]. This phenomenon can manifest across multiple relational contexts. It is frequently observed in intimate or romantic partnerships [3]–[5], but it can also arise within professional environments, including relationships between employers and employees or among colleagues [6],[7]. Furthermore, gaslighting has been identified in healthcare settings, where power imbalances may facilitate manipulative dynamics between clinicians, such as physicians or nurses, and their patients [8]–[10].

Nurses continue to face substantial challenges while navigating an already overburdened healthcare environment [11]–[14]. Numerous factors, such as the consequences of the COVID-19 pandemic, excessive workload, inadequate leadership, workplace bullying, and persistent emotional exhaustion, hinder their ability to perform their duties effectively [15]–[17]. These conditions not only compromise nurses' clinical performance but also adversely affect their psychological well-being, professional commitment, and the quality of care they provide to patients [18]–[20]. Within this highly stressful and demanding context, nurses are now increasingly confronted with yet another work-related problem, i.e., workplace gaslighting.

Workplace gaslighting may arise within professional relationships characterized by power asymmetries and hierarchical inequities. Individuals who occupy positions of authority and engage in gaslighting behaviors exhibit substantial leadership deficiencies, relying on the manipulation of social interactions, the dissemination of misinformation, and the deliberate cultivation of confusion and self-doubt among subordinates rather than exercising ethical and supportive leadership practices [21]. Within this context, workplace gaslighting perpetrated by nurse managers against staff nurses represents a significant threat to nurses' psychological well-being, professional functioning, and overall work life. Since the phenomenon of workplace gaslighting in nurses is new, there are only a few studies in this research field [22]–[25]. In particular, literature suggests the negative association between workplace gaslighting and nurses' agility [23], career entrenchment [24], quality of care [25], and patient safety [25]. Moreover, workplace gaslighting among nurses is associated with increased quiet quitting [25], job burnout [22], and turnover intention [22]. In other words, workplace gaslighting may affect not only nurses' careers but also the quality of healthcare services.

Given the limited availability of empirical research specifically addressing workplace gaslighting among nurses, we deemed it necessary to draw upon studies examining the effects of toxic leadership on staff nurses. Integrating this broader body of evidence strengthens our theoretical rationale by providing a conceptual and empirical foundation for understanding how gaslighting, as a form of harmful interpersonal and supervisory behavior, may influence the outcomes in our study. We examined studies on toxic leadership, since workplace gaslighting and toxic leadership represent two forms of organizational dysfunction that undermine employee psychological safety and impede healthy organizational functioning. Although the constructs may overlap—particularly when gaslighting is deployed as a tactic by destructive leaders—they are theoretically and empirically distinct phenomena [26],[27]. Toxic leadership represents a broader, systemic pattern of destructive managerial behavior that negatively affects both employees and organizational functioning. While gaslighting may be one tactic within a toxic leader's behavioral repertoire, not all toxic leaders engage in gaslighting, and gaslighting does not require formal authority to occur [28],[29]. Several studies show a negative association between toxic leadership and nurses' mental health including mental health variables such as psychological distress, psychological well-being, psychological health, quality of life, satisfaction with health, and social relationships [30]–[33]. Additionally, the negative influence of toxic leadership on nurses' work-related variables is well-known including variables such as job satisfaction, work engagement, quality of work life, productivity, organizational commitment, conflict management, intention to stay, organizational trust, and turnover intention [31],[32],[34]–[40]. Furthermore, toxic leadership can affect the quality of healthcare services by threatening patient safety, organization performance, and the quality of care, and by increasing nurse-reported adverse events [41]–[43].

In this context, the assessment of gaslighting consequences is crucial to improve nurses' work life and productivity. Thus, we examined the association between workplace gaslighting and nurses' mental health and work life.

## 2. Methods

### 2.1. Study design

We conducted a cross-sectional study in Greece. We collected our data through an online survey during December 2024. We created an online version of the study questionnaire with Google forms, and then we posted it in nurses' groups on Facebook and Instagram. Moreover, we sent the questionnaire in nurses' LinkedIn profiles through inbox messages. To minimize the possibility of duplicate responses, potential participants were informed in the introductory note that individuals who had already taken part in the study should not complete the questionnaire a second time. Thus, we obtained a convenience sample. Participants were required to meet the following criteria: 1) They are a clinical nurse in a healthcare facility, 2) they are a staff nurse and not a supervisor, 3) they have a minimum of one year of work experience, and 4) they agree to participate in our study. We applied the Strengthening the Reporting of Observational Studies in Epidemiology (STROBE) guidelines in our study [44].

We included one predictor (workplace gaslighting) and four confounders (gender, age, educational level, and work experience) in our models. Thus, considering an anticipated effect size of 0.04 between workplace gaslighting and outcomes (anxiety-like symptoms, depressive-like symptoms, quiet quitting, and work engagement), a statistical power of 95%, and a margin of error of 5%, the sample size was estimated to be 327 nurses. We used G*Power v.3.1.9.2 to calculate our sample size.

### 2.2. Measurements

We used the Gaslighting at Work Scale (GWS) [45] to measure levels of workplace gaslighting among our nurses. The GWS includes 11 items, e.g., “In the last six months, your supervisor denied saying things that you remember him/her saying”, “In the last six months, your supervisor lied to you”, and “In the last six months, your supervisor made you depend on him/her for making decisions about your work”. The GWS includes two factors, namely “loss of self-trust” (five items) and “abuse of power” (six items). Answers are on a five-point Likert scale; never (1), rarely (2), sometimes (3), very often (4), and always (5). The score on the GWS is calculated as an average of all answers. The score on the two factors is calculated similarly. Thus, the scores for the GWS and the two factors range from 1 to 5. Higher values indicate higher levels of gaslighting behaviors from supervisors. We used the Greek version of the GWS [45]. In our study, Cronbach's alpha for the GWS was 0.939. Moreover, Cronbach's alpha for the factors “loss of self-trust” and “abuse of power” was 0.900 and 0.906, respectively.

To assess anxiety-like symptoms and depressive-like symptoms in our sample, we employed the Patient Health Questionnaire-4 (PHQ-4) [46]. This tool comprises four items: two for anxiety-like symptoms and two for depressive-like symptoms. It should be noted that the PHQ-4 assesses anxiety-like and depressive-like symptoms, rather than actual levels of anxiety and depression. Responses are recorded on a four-point Likert scale ranging from 0 (not at all) to 3 (nearly every day). The scores for both anxiety-like symptoms and depressive-like symptoms scales span from 0 to 6, with higher scores indicating more severe symptoms. We utilized the validated Greek version of the PHQ-4 [47]. In our study, Cronbach's alpha coefficients for the “anxiety-like symptoms” and “depressive-like symptoms” factors were 0.782 and 0.818, respectively.

To evaluate quiet quitting among our nurses, we implemented the Quiet Quitting Scale (QQS) [48]. This tool consists of nine items (e.g., “I do the basic or minimum amount of work without going above and beyond”, “I take as many breaks as I can”, and “If a colleague can do some of my work, then I let him/her do it”), with responses recorded on a five-point Likert scale ranging from strongly disagree/never (1) to strongly agree/always (5). The QQS includes three factors, i.e., “detachment” (four items), “lack of initiative” (three items), and “lack of motivation” (two items). The score for each factor is calculated as the mean of responses to the factor items, resulting in a range from 1 to 5. Higher scores signify increased levels of quiet quitting. We used the validated Greek version of the QQS [49]. In our study, the Cronbach's alpha for the QQS was 0.851. Moreover, Cronbach's alpha for the factors “detachment”, “lack of initiative”, and “lack of motivation” was 0.805, 0.761, and 0.807, respectively.

To measure work engagement in our sample, we applied the Utrecht Work Engagement Scale-3 (UWES-3) [50]. This tool is composed of three items (e.g., “At my work, I feel bursting with energy”), with responses recorded on a seven-point Likert scale ranging from never (0) to everyday (6). The mean score on the UWES-3 spans from 0 to 6, with higher values indicating greater work engagement. We employed the validated Greek version of the UWES-3 [51]. In our study, Cronbach's alpha for the UWES-3 was 0.812.

Moreover, we measured several demographic characteristics such as gender (females or males), age (continuous variable), MSc/PhD diploma (no or yes), and work experience (continuous variable).

### 2.3. Ethical issues

We conducted our study in accordance with the Declaration of Helsinki [52]. In addition, the Ethics Committee of the Faculty of Nursing, National and Kapodistrian University of Athens, approved our study protocol (approval number: 15, December 9, 2024). We collected our data on an anonymous and voluntary basis. We informed participants about the aim and the design of our study, and they gave their informed consent. Moreover, we informed participants that the PHQ-4 is a screening instrument for mental health problems and that elevated scores may be indicative of anxiety or/and depression disorders. Accordingly, nurses who obtained high PHQ-4 scores were advised to seek further evaluation from mental health services, as they may belong to a high-risk group for developing anxiety or/and depressive disorders.

### 2.4. Statistical analysis

We presented categorical variables as numbers and percentages. Also, we used mean, standard deviation (SD), median, and interquartile range to present continuous variables. We used the Kolmogorov-Smirnov test and Q-Q plots to examine the distribution of continuous variables. We found that continuous variables followed a normal distribution. Moreover, we considered demographic variables (gender, age, educational level, and work experience) as potential confounding factors. Thus, we performed simple and multivariable linear regression analysis to identify the association between workplace gaslighting, anxiety-like symptoms, depressive-like symptoms, quiet quitting, and work engagement. In this case, workplace gaslighting was the independent variable, while anxiety-like symptoms, depressive-like symptoms, quiet quitting, and work engagement were the dependent variables. First, we performed simple linear regression analysis, and then we constructed a final multivariable model by eliminating confounders to estimate the independent effect of gaslighting on anxiety-like symptoms, depressive-like symptoms, quiet quitting, and work engagement. The two factors (i.e., “loss of self-trust” and “abuse of power”) of the GWS were highly correlated (Pearson's correlation coefficient = 0.791, p-value < 0.001). Similarly, age and work experience were highly correlated (Pearson's correlation coefficient = 0.940, p-value < 0.001). Moreover, when we inserted these high correlated variables simultaneously in the multivariable models, we recognized multicollinearity issues. In particular, we examined the variance inflation factors of the independent variables in the multivariable models. When we included the “loss of self-trust” scale, “abuse of power” scale, age, and work experience, the variance inflation factors were higher than 8, indicating multicollinearity issues since the acceptable values are lower than 5 [53]. Thus, we included the total score on the GWS instead of its two factors to avoid multicollinearity issues. Also, we included work experience and not age in the multivariable models. We presented unadjusted and adjusted beta coefficients, 95% confidence intervals (CI), and p-values. P-values less than 0.05 were considered statistically significant. We used an IBM SPSS 28.0 (IBM Corp. Released 2021. IBM SPSS Statistics for Windows, Version 28.0. Armonk, NY: IBM Corp) for the analysis.

## 3. Results

### 3.1. Demographic characteristics

Demographic characteristics of nurses are shown in Table 1. The study population included 369 nurses. Most of them were females (85.9%). The mean age of nurses was 37.86 years (SD: 10.44) with a median age of 37 years (interquartile range: 16). Six out of ten nurses possessed a MSc/PhD diploma (61.5%). Mean work experience was 13.74 years (SD: 10.19) with a median of 12 years (interquartile range: 15).

**Table 1.** Demographic characteristics of nurses (N = 369).

Characteristics	N	%
Gender		
Males	52	14.1
Females	317	85.9
Age (years)^a^	37.86	10.44
MSc/PhD diploma		
No	142	38.5
Yes	227	61.5
Work experience (years)^a^	13.74	10.19

Note: ^a^ mean, standard deviation.

### 3.2. Study scales

Descriptive statistics for the study scales are shown in Table 2. The mean score on the GWS was 2.58 (SD: 0.96), while on the factors “loss of self-trust” and “abuse of power”, it was 2.27 and 2.85, respectively. Given the range of the GWS (1–5), the theoretical midpoint of the scale is 3.0. Thus, the mean score of 2.58 suggested that our nurses experienced a moderate level of gaslighting behaviors from supervisors. Moreover, abuse of power was more frequent than loss of self-trust in our sample. Considering that the anxiety-like symptoms scale and the depressive-like symptoms scale range from 0 to 6, and that the theoretical midpoint of the scales is 3, our findings showed moderate anxiety-like symptoms and depressive-like symptoms since the mean score was 2.89 and 2.76, respectively. Additionally, the levels of quiet quitting were moderate in our nurses since the mean score on the QQS was 2.43, while the range of the scale is 1–5 with a theoretical midpoint of 3.0. Our nurses experienced more frequent lack of motivation (mean: 2.83) than lack of initiative (mean: 2.45) and detachment (mean: 2.22). Finally, our nurses showed moderate work engagement since the mean score on the UWES-3 was 3.46 (SD: 1.49), while the scale ranges from 0 to 6 and has a theoretical midpoint of 3.0.

**Table 2.** Descriptive statistics for the study scales (N = 369).

Scale	Mean	Standard deviation	Median	Interquartile range
Gaslighting at Work Scale	2.58	0.96	2.55	1.59
Loss of self-trust	2.27	0.97	2.20	1.60
Abuse of power	2.85	1.04	2.83	1.67
Patient Health Questionnaire-4	5.65	3.10	5.00	4.00
Anxiety-like symptoms	2.89	1.62	3.00	2.00
Depressive-like symptoms	2.76	1.70	2.00	2.00
Quiet Quitting Scale	2.43	0.71	2.33	0.89
Detachment	2.22	0.80	2.00	1.00
Lack of initiative	2.45	0.91	2.33	1.33
Lack of motivation	2.83	0.98	2.50	1.50
Utrecht Work Engagement Scale-3	3.46	1.49	3.67	2.67

### 3.3. Association between workplace gaslighting, anxiety-like symptoms, and depressive-like symptoms

We found a positive association between workplace gaslighting, anxiety-like symptoms, and depressive-like symptoms in our nurses (Table 3). After adjusting for gender, age, educational level, and work experience, we found a positive association between the score on the GWS and anxiety-like symptoms (b = 0.758, 95% CI = 0.606 to 0.909, p < 0.001), as well as with depressive-like symptoms (b = 0.720, 95% CI = 0.555 to 0.885, p < 0.001). In other words, nurses who experienced higher levels of gaslighting behaviors from their supervisors had also more anxiety-like symptoms and depressive-like symptoms. In practical terms, a one-unit increase in the GWS corresponded to an estimated 0.758-unit increase in anxiety-like symptoms, and a 0.720-unit increase in depression-like symptoms, after adjusting for the other variables included in the multivariable model.

**Table 3.** Linear regression models with anxiety-like symptoms and depressive-like symptoms as the dependent variables, and the score on the Gaslighting at Work Scale as the independent variable (N = 369).

Dependent variable	Univariate models			Multivariable model^a^		
	Unadjusted beta coefficient	95% CI for beta	P-value	Adjusted beta coefficient	95% CI for beta	P-value
Anxiety-like symptoms^b^						
Score on GWS	0.765	0.609 to 0.920	<0.001	0.758	0.606 to 0.909	<0.001
Depressive-like symptoms^c^						
Score on GWS	0.758	0.606 to 0.909	<0.001	0.720	0.555 to 0.885	<0.001

Note: ^a^ Multivariable models are adjusted for gender, age, educational level, and work experience; ^b^ R^2^ for the multivariable model = 25.9%, p-value for ANOVA < 0.001; ^c^ R^2^ for the multivariable model = 20.1%, p-value for ANOVA < 0.001; CI: confidence interval; GWS: Gaslighting at Work Scale.

### 3.4. Association between workplace gaslighting, quiet quitting, and work engagement

Table 4 shows the results from the linear regression analysis with quiet quitting and work engagement as the dependent variables. After eliminating confounding factors, we found a positive association between the score on the GWS and quiet quitting (b = 0.258, 95% CI = 0.186 to 0.330, p < 0.001). Moreover, we found a negative association between the score on the GWS and work engagement (b = −0.353, 95% CI = −0.512 to −0.195, p < 0.001). In other words, workplace gaslighting was associated with quiet quitting and work engagement among our nurses. In practical terms, a one-unit increase in the GWS corresponded to an estimated 0.258-unit increase in quiet quitting, and a 0.353-unit decrease in work engagement, after adjusting for the other variables included in the multivariable model.

**Table 4.** Linear regression models with quiet quitting and work engagement as the dependent variables, and the score on the Gaslighting at Work Scale as the independent variable (N = 369).

Dependent variable	Univariate models			Multivariable model^a^		
	Unadjusted beta coefficient	95% CI for beta	P-value	Adjusted beta coefficient	95% CI for beta	P-value
Quiet quitting^b^						
Score on GWS	0.262	0.190 to 0.333	<0.001	0.258	0.186 to 0.330	<0.001
Work engagement^c^						
Score on GWS	−0.353	−0.513 to −0.201	<0.001	−0.353	−0.512 to −0.195	<0.001

Note: ^a^ Multivariable model is adjusted for gender, age, educational level, and work experience; ^b^ R^2^ for the multivariable model = 13.3%, p-value for ANOVA < 0.001; ^c^ R^2^ for the multivariable model = 4.8%, p-value for ANOVA < 0.001; CI: confidence interval; GWS: Gaslighting at Work Scale.

## 4. Discussion

The role of nurses is fundamental to the effective functioning of healthcare systems. Consequently, it is imperative to remain vigilant regarding factors that may jeopardize nurses' well-being, particularly those related to occupational conditions and mental health. Although workplace gaslighting remains an understudied phenomenon, emerging evidence suggests that it constitutes a significant threat to nurses' psychological well-being and professional performance. In this context, the present study was deemed essential to assess the prevalence of workplace gaslighting experienced by nurses and to examine its association with anxiety-like and depressive-like symptoms. Furthermore, this study sought to investigate the association between workplace gaslighting, quiet quitting, and work engagement among nurses. By exploring these associations, our study aims to contribute to a more comprehensive understanding of how gaslighting manifests in nursing contexts and how it may influence nurses' mental health and work life.

Our sample demonstrated moderate levels of anxiety-like and depressive-like symptoms. Such findings align with previous research suggesting that nurses frequently experience comparable levels of psychological distress due to demanding work environments and sustained occupational pressures [14],[54]–[56]. In addition, our sample reported moderate levels of quiet quitting and work engagement. These findings are consistent with prior research indicating that nurses exhibit a higher propensity for quiet quitting compared to other professional groups, often driven by detrimental workplace conditions. Empirical studies have shown that factors such as workplace gaslighting and mobbing, bullying, burnout, and inadequate supervisory support contribute significantly to nurses' turnover intentions and disengagement from their roles [54],[57]–[59].

Furthermore, our sample exhibited a moderate lack of motivation. This finding is consistent with evidence indicating that nurses who experience diminished motivation are more likely to express turnover intentions and engage in quiet quitting behaviors. Such patterns have also been documented in previous studies, where similar workplace stressors and adverse organizational conditions have been shown to contribute to decreased motivation and a heightened likelihood of disengagement among nurses [11],[15],[59],[60].

In our sample, we found a positive association between workplace gaslighting, anxiety-like symptoms, and depression-like symptoms. To the best of our knowledge, the association between workplace gaslighting, anxiety, and depression has not yet been investigated in nurses. However, a recent study involving white-collar employees provides support for our findings, demonstrating that individuals who experience higher levels of workplace gaslighting also report elevated levels of anxiety-like and depressive-like symptoms [61]. This evidence reinforces the association observed in our sample and aligns with emerging research indicating the potential psychological impact of gaslighting across occupational groups. Additionally, several studies have demonstrated a negative association between toxic leadership and nurses' mental health, documenting adverse outcomes across multiple psychological domains. These include heightened psychological distress, reduced psychological well-being, diminished overall psychological health, lower quality of life, decreased satisfaction with personal health, and impaired social relationships [30]–[33]. Workplace gaslighting constitutes a covert but deeply harmful form of psychological manipulation, and it could have a substantial negative impact on nurses' mental health, motivation, and overall occupational well-being. Gaslighting behaviors, such as trivializing concerns, distorting information, denying facts, and undermining self-confidence, create a destabilizing work environment in which nurses gradually begin to doubt their own judgment, competence, and perception of reality. Thus, psychological resilience and professional functioning may deteriorate markedly among nurses that experience such manipulation. Moreover, gaslighting behavior may act as a hidden driver of emotional exhaustion and burnout among nurses by eroding psychological safety and creating sustained emotional distress. Such outcomes are particularly concerning for nurses, whose roles involve high emotional labor and continuous exposure to stressful clinical environments.

Moreover, our multivariable analysis showed an association between workplace gaslighting, quiet quitting, and work engagement. In particular, we found a positive association between workplace gaslighting and quiet quitting. On the other hand, we found a negative association between workplace gaslighting and work engagement. Literature supports our findings since several studies show a negative association between workplace gaslighting and work-related variables, such as nurses' agility [23] and career entrenchment [24]. Additionally, there is evidence that workplace gaslighting in nurses may also affect quality of care and patient safety [25]. Additionally, the detrimental impact of toxic leadership on nurses' work-related outcomes is well-documented in the literature. Empirical studies consistently demonstrate that toxic supervisory behaviors are associated with decreased job satisfaction, reduced work engagement, diminished quality of work life, lower productivity, weakened organizational commitment, and impaired conflict management processes. Moreover, toxic leadership has been shown to erode organizational trust, decrease nurses' intention to remain in their positions, and substantially increase turnover intention [31],[32],[34]–[40]. It seems to be that manipulated nurses become increasingly detached from their roles and responsibilities. Thus, nurses who experience high levels of gaslighting may also experience reduced motivation and diminished capacity to remain engaged in their work. This pattern is reinforced by the fact that gaslighting undermines work motivation and reduces job embeddedness, thereby creating conditions that heighten turnover intentions among nurses. Moreover, gaslighting contributes to a breakdown of trust in professional relationships, which is essential for effective teamwork in clinical practice. By fostering confusion and self-doubt, gaslighting disrupts nurses' ability to advocate for themselves, collaborate confidently with colleagues, and make autonomous clinical decisions.

Our study had several limitations. First, we conducted a cross-sectional study, and thus, we cannot infer a causal relationship between workplace gaslighting, anxiety-like symptoms, depressive-like symptoms, quiet quitting, and work engagement. Second, although we covered the minimum requirement for the sample size, the employment of a convenience sample introduced selection bias in our study. For instance, our sample comprised mainly of females with a MSc/PhD diploma. Furthermore, since we recruited our convenience sample through social media platforms, a selection bias is probably introduced. Our sample may have included nurses who are more active on social media or more interested in work-related issues. In other words, overrepresentation of more engaged or sensitized nurses is quiet probable in our study. Therefore, the representativeness of our sample is uncertain, and any generalization of our findings should be made with considerable caution, even when referring to nurses in Greece. Thus, there is a need for studies with random and more representative samples of nurses. Additionally, since we performed our study in a European country, future studies should be conducted in different countries and cultural settings to further examine the association between workplace gaslighting, anxiety-like symptoms, depressive-like symptoms, quiet quitting, and work engagement. Third, we eliminated only five confounders (i.e., gender, age, educational level, and work experience) in our analyses. We used multivariable models to eliminate these confounders in our study but several other variables can also act as confounders, such as work in the public or private domain, clinical settings, and personality traits of supervisors or staff nurses. Moreover, several work-related variables such as job burnout, job satisfaction, and work environment could act as potential confounders in the association between workplace gaslighting, anxiety-like symptoms, depressive-like symptoms, quiet quitting, and work engagement. Future studies should eliminate more confounders to further validate our findings. Furthermore, we should notice that we used a screening tool, i.e., the PHQ-4, to assess levels of anxiety-like symptoms and depressive-like symptoms. Therefore, we did not measure anxiety and depression in our participants. Further studies could minimize this bias by performing clinical examinations and identifying levels of anxiety and depression through more objective evaluations. Moreover, we should recognize that workplace gaslighting is inherently based on subjective perceptions and is expressed through self-reported data in our study. Literature suggests that unstructured nursing documentation can vary substantially in terminology and meaning, influencing the interpretation and standardization of complex phenomena in clinical settings [62]. Therefore, since workplace gaslighting is a new work-related issue, future studies should further clarify this concept by taking into account how subjective perceptions are linguistically expressed and operationalized. Finally, we used valid instruments (i.e., GWS, PHQ-4, QQS, and UWES-3) to measure mental health and work life of our nurses. However, information bias is probable in our study due to the self-nature of these instruments.

## 5. Conclusions

Our results suggest that gaslighting is associated with higher levels of anxiety-like symptoms and depressive-like symptoms among nurses. Furthermore, our study shows a negative association between workplace gaslighting and work engagement, and a positive association between gaslighting and quiet quitting. Gaslighting is associated with poor mental health and alarming consequences for employees' commitment to their jobs. Nurses' roles are vital for healthcare systems and high-quality health services. Yet, nurses are constantly facing a number of adversities in their day-to-day jobs. It is vital that policymakers and nursing managers should look into the matter in order to secure a healthy work environment and protect nurses from aggravating circumstances that may affect their mental health and work engagement.
